# Standard Flow Multiplexed Proteomics (SFloMPro)—An Accessible Alternative to NanoFlow Based Shotgun Proteomics

**DOI:** 10.3390/proteomes10010003

**Published:** 2022-01-13

**Authors:** Benjamin C. Orsburn, Sierra D. Miller, Conor J. Jenkins

**Affiliations:** 1 Department of Pharmacology and Molecular Sciences, The Johns Hopkins University School of Medicine, Baltimore, MD 21205, USA; 2 Biology Department, Millersville University, Millersville, PA 17551, USA; sierradesireemiller@gmail.com; 3 Department of Chemistry and Biochemistry, University of Maryland, College Park, MD 20737, USA; conor.jenkins@outlook.com

**Keywords:** proteomics, isobaric labeling, TMT, high-flow proteomics

## Abstract

Multiplexed proteomics using isobaric tagging allows for simultaneously comparing the proteomes of multiple samples. In this technique, digested peptides from each sample are labeled with a chemical tag prior to pooling sample for LC-MS/MS with nanoflow chromatography (NanoLC). The isobaric nature of the tag prevents deconvolution of samples until fragmentation liberates the isotopically labeled reporter ions. To ensure efficient peptide labeling, large concentrations of labeling reagents are included in the reagent kits to allow scientists to use high ratios of chemical label per peptide. The increasing speed and sensitivity of mass spectrometers has reduced the peptide concentration required for analysis, leading to most of the label or labeled sample to be discarded. In conjunction, improvements in the speed of sample loading, reliable pump pressure, and stable gradient construction of analytical flow HPLCs has continued to improve the sample delivery process to the mass spectrometer. In this study we describe a method for performing multiplexed proteomics without the use of NanoLC by using offline fractionation of labeled peptides followed by rapid “standard flow” HPLC gradient LC-MS/MS. Standard Flow Multiplexed Proteomics (SFloMPro) enables high coverage quantitative proteomics of up to 16 mammalian samples in about 24 h. In this study, we compare NanoLC and SFloMPro analysis of fractionated samples. Our results demonstrate that comparable data is obtained by injecting 20 µg of labeled peptides per fraction with SFloMPro, compared to 1 µg per fraction with NanoLC. We conclude that, for experiments where protein concentration is not strictly limited, SFloMPro is a competitive approach to traditional NanoLC workflows with improved up-time, reliability and at a lower relative cost per sample.

## 1. Introduction

Shotgun proteomics has several technical challenges that currently inhibits its widespread adoption. Many of these have been addressed by recent advances in mass spectrometry engineering leading to marked increases in accuracy, resolution, speed, and sensitivity. Both instrument data acquisition and post processing software have improved, with the transition of modern algorithms into graphic user interfaces (GUIs) [1,2,3,4].

A major challenge in shotgun proteomics is the use of nanoflow liquid chromatography (NanoLC). In LC-MS based shotgun proteomics samples are diluted by chromatography running buffer. Due to the relatively low sensitivity of the first tandem mass spectrometers utilized for global proteomics, concentrating the sample with the use of progressively lower flow rates was an attractive solution [5]. The resulting methodology, NanoLC, was rapidly adopted in shotgun proteomics, to the point that it has been recently and accurately described as dogma [6]. 

NanoLC requires the precise plumbing of fragile fused silica columns with internal diameters ranging 20–100 µm and flow rates typically ranging 20–500 nL per minute. As few pumps existed that could reliably deliver these flow rates, early practitioners utilized solvent splits from higher flow pumps that would divert as much as 99% of the utilized solvent to waste with the remaining 1% used for peptide gradient elution [7]. 

Recent advances have seen the utilization of pressurized gas-driven, low-capacity syringe pumps that can directly construct reversed phase chromatography gradients of these flow rates without solvent splits. Furthermore, the fused silica lines are now often coated in plastic sheaths and integrate “zero dead volume” union connections that simplify the plumbing of NanoLC systems. 

In order to work with nL/min flow rates, even modern systems require more maintenance to prevent and remove the presence of trapped atmospheric air and evaporated solvents when compared to conventional flow technologies. The user manual for the NanoLC system recommends daily cycles of solvent purges and programs to flush air from pumps (vendor user manual). In contrast, an ultra-high pressure (UHPLC) system purchased at this same date from the same vendor recommends solvent flushes at monthly intervals (vendor user manual). Despite this increase in maintenance, the NanoLC is orders of less reproducible than higher flow LC systems. UHPLC conventional flow systems have shown peak retention times varying less than one second over hundreds of samples [1]. It is well accepted that NanoLC experiments have less reproducible retention times [8]. The recently described IonStar system for clinical proteomics carefully controls all chromatography variables for maximum NanoLC reproducibility using 100 cm columns, but still requires adjustments to align retention times that differ by as much as 60 s between runs [9].

Common quantitative proteomic experiments utilize isobaric tags such as the commercial iTRAQ and TMT products [10]. By performing relative quantification in the MS/MS spectra of tagged peptides, peptide retention times and NanoLC retention time reproducibility are less of a concern. In this procedure, peptides from independent samples are each labeled with a unique isobaric tag and then combined for LCMS. The relative abundances of the peptides are revealed in MS/MS or MS/MS/MS fragmentation spectra and sample to sample quantitation can be achieved. A critical step in the experiment to minimize bias is the efficiency of the peptide labeling. In order to obtain complete labeling of the peptides, the manufacturer of the reagents recommends a ratio of 1:8 peptide to label. 

However, recent studies have demonstrated effective complete labeling of peptides with a lower ratio of peptides, with 1:4 and even 1:2 shown to be effective [11,12]. Currently, the smallest aliquot commercially available for each labeling compound is 800 µg of label per sample. This appears to constitute a large discrepancy, as the typical upper limit for peptides for NanoLC separation, is considered 0.2–4 µg of peptide injection [13]. This results in the generation of excess materials in either a large amount of unused label or labeled peptide. In addition, due to the high reactivity of the label, these excess materials are often considered unsuitable for use following rehydration and are typically discarded as waste [11]. If isobaric reagents are aliquoted and dehydrated in facilities operating with high relative efficiency, as in our respective labs, we will label an excess of peptides for analysis as it is more efficient to have extra sample prepared in case of failed instrument QCs rather than to reproduce the entire labeling process. In either case, we find it difficult if not impossible to fully utilize every microgram of reagent present in a commercial reagent kit. 

Alternative approaches to NanoLC are gaining in popularity, with multiple studies using capillary zone electrophoresis [14,15] and microflow chromatography [16,17] demonstrating promise as alternative methodologies. 

Recent work has also described the use of “standard flow” proteomics, reaching the conclusion that flow rates in the 50–200 µL per minute range can provide quality proteomics data with proper optimization [6,18]. Building on this work, we describe a standard flow multiplexed proteomics (SFloMPro) workflow. Our results show that SFloMPro produces comparable data to that of NanoLC but requires 20 times more labeled peptide on column. Due to the rapid preparation of LC gradients and sample loading of conventional flow uHPLC systems, we demonstrate a time savings of nearly 25% over our in house NanoLC solutions, with no changes in our sample preparation workflow. By removing the requirements of the purchase of a NanoLC system and the technical hurdles associated with this technology, SFloMPro requires only a HPLC-Orbitrap instrument configuration to perform quantitative proteomics. 

Using SFloMPro, we performed multiplexed quantification of over 8000 mammalian proteins in approximately 24 h of total instrument acquisition time. We conclude that SFloMPro is an accessible alternative for high throughput proteomics in conditions such as cell culture or any biological samples where sample abundance is not a strictly limiting factor. 

## 2. Materials and Methods

### 2.1. Cells and Cell Culture 

One T25 flask of murine cell culture line BalbC was prepared per condition. Cells were harvested lysed, resulting in 1–4 mg total protein per condition, as quantified by BCA. Approximately 200 µg of protein was utilized for digestion with reduction and alkylation of the cysteines with DTT and iodoacetamide, respectively. Six channels were labeled with TMT 11-plex reagent in a 1:4 ratio of peptide to label. The following channels were utilized in this study: 129N,129C, 130N, 130C, 131N, 131C, where the last channel is pooled samples.

### 2.2. High pH Reversed Phase Fractionation

The combined labeled peptides were separated on a Thermo Accela 1250 HPLC using a Waters XBridge BEH130 C18 3.5 µm 2.1 mm × 150 mm column using a flow rate of 0.2 mL/min with Buffer A and B as 25 mM Ammonium bicarbonate pH 8.0 in LC-MS grade water and LC-MS grade acetonitrile (Fisher), respectively. Peptides were separated on a linear gradient of 5–35% B over 60 min, with a linear increase to 70% B over 12 min. Fractions were collected every 45 s using a Foxy Junior fraction collector to result in complete filing of a 96 well plate. Stepwise concatenation was performed by incrementally combining every 24th well, for example: sample 1 is combined results of wells 1, 25, 49, and 73 combined; sample 2 is combined result of wells 2, 26, 50, and 74. The 24 resulting fractions were subjected to one final clean up with Pierce peptide spin columns (part number 89851) and SpeedVac to near dryness for LC-MS analysis.

### 2.3. NanoLC Separation

Approximately 1 µg of peptides were loaded by EasyNLC 1000 (Thermo Fisher, Bremen, Germany) onto a 3 mm PepMap desalting column prior to solvent loading gradient elution on the 15 cm EasySpray 3 µm PepMap column. Buffers consisted of 0.1% formic acid in LCMS grade water (A) and 80% LCMS grade acetonitrile (B) from Thermo. Prior to each injection the pre-column was equilibrated with 1 µL Buffer A at a maximum pressure of 600 bar. The analytical column was equilibrated the same with 6 µL Buffer A. 

For each injection, 2 µL of sample was picked up and a total of 6 µL of sample plus loop loading buffer was loaded to the trap column prior to closing the waste valve and beginning the gradient. The gradient consisted of a two separate stages: Stage 1 began with a 95% Buffer A/5% B at 300 nL/min that increased to 24% B in 31 min, followed by an increase to 38% B by 56 min. The column was reconditioned by increasing the flow rate to 500 nL/min and ramping to 98% B by 65 min before column equilibrations for the next sample. 

### 2.4. Standard-Flow HPLC Separation and Ionization Conditions

All analyses used a Vanquish H uHPLC (Thermo) coupled to Q Exactive mass spectrometer. Starting conditions were based on a recent report by Lenco et al. [6] using a Waters BEH Peptide BEH C18 1.7 µm × 2.1 × 150 mm column. Optimization of injection and gradient was performed using the HeLa peptide standard (Pierce) and ranged from a starting material of 500 nanogram to 10 microgram total peptide. The final gradient utilized 5% DMSO and 0.1% formic acid with Milli-Q water as Buffer A and the same buffer additives in HPLC grade acetonitrile as Buffer B. The gradient began at 200 µL/min 0% B and ramped to 5% B in 5 min followed by an increase to 30% B by 50 min, an increase to 90% B in 6 min with increase to 0.4 mL/min for the remainder of the run, with a 1 min hold before resuming to baseline conditions to a total run length of 65 min. All gradient changes were with a pump curve of 5 arbitrary units. The Q Exactive Classic system was equipped with HESI-II system (Thermo) and used the following source conditions described by the Xcalibur Tune software for 0.2 mL/min flow rates: ESI voltage 3500, capillary temperature 325, sheath gas 45, auxiliary gas 10, spare gas 2, probe heater temperature of 150C and S-lens RF of 70.

### 2.5. Mass Spectrometer Conditions

An identical data-dependent acquisition method was used for both experiments. MS1 spectra were acquired at a resolution of 70,000 with an AGC target of 3 × 10^6^ charges or 100 ms maximum ion injection time. MS1 was acquired 381–1581 *m*/*z*. The top 10 ions were selected for MS/MS using a resolution of 35,000 at *m*/*z* of 200 with an AGC target of 1 × 10^6^ or maximum ion injection time of 114 ms. An isolation window of 2.2 Da was used along with a fixed first mass of 110 *m*/*z*. A normalized collision energy of 30 was used for both experiments. Ions of unassigned, single charge, or charge state of 8 or above were excluded from MS/MS and dynamic exclusion was used with a 30 s window.

### 2.6. Data Processing

All data was processed in Proteome Discoverer 2.4 (Thermo). For TMT quantitative analysis the manufacturer default workflow for reporter ion quantification for TMT 11-plex reagent. The SequestHT search engine was used to search the data against the UniProt reviewed database downloaded on 14 April 2021 and the cRAP contaminants database (The Global Proteome Machine, www.gpm.org, accessed 1 February 2021) with the following settings: 10 ppm MS1 tolerance, 0.02 Da MS/MS tolerance, variable modifications of methionine oxidation and acetylation of the protein N terminus, along with static modifications of carbamidomethylation of cysteines and the addition of the TMT 6/10/11 plex tag on peptide N-terminus and lysine. Up to two missed cleavage events were allowed. To evaluate relative peak widths and other variables the samples were reran as described, with the removal of the reporter ion quantification node and replacement with the Minora feature alignment and peak detection node using default parameters. To correlate results between the nanoflow LCMS experiments and the high flow experiments, the data was processed in MaxQuant [19] 1.6.17.0 and using the default Orbitrap TMT parameters with the databases described above. Perseus 1.6.15.0 was used for downstream analysis [20]. For generation of the Pearson correlation comparisons between each channel, the normalized reporter intensity values were imported. The data was filtered to remove decoy protein groups as well as all entries identified exclusively by site. The values were log transformed and imputation was performed using by replacing missing values from normal distribution. The resulting values for the 6 labeled channels utilized in the study were compared by multi-scatter plot and Pearson correlation coefficient was displayed in each ratio comparison. 

## 3. Results and Discussion

### 3.1. Total Instrument Acquisition Time

In the increasingly competitive research core environment the number of samples completed per unit time may be a critical measurement for financial success [21]. A recent study that utilized high levels of high pH offline and rapid NanoLC gradients noted the minimum loading time of 22 min for a nanoflow LC system similar to the one employed in this study [22]. In an attempt to keep variable consistent the total acquisition time for the NanoLC and SFloMPro samples were set at approximately 60 min. However, the time stamps embedded in the manufacturer binary files indicates that the 24 NanoLC samples required 33.5 h from first to final sample. In contrast, the 24 SFloMPro samples were only separated by 25.3 h, a 25% reduction in run time. While multiple approaches have been demonstrated for parallel trapping and elution in NanoLC proteomics to increase throughput, the systems in our respective labs do not have these capabilities [23,24].

### 3.2. Peptide and Protein Identifications

Table 1 is a summary of the two experiments. Full results are available in the combined Supplemental. Although the NanoLC experiment acquired more total MS/MS spectra and peptide spectral matches (PSMs), this did not translate to a corresponding increase in the number of peptides and proteins identified. An average of 2.1 PSMs were identified for each peptide in the NanoLC files, compared to 1.4 PSMs in the SFloMPro file set (Supplemental S2). The mass spectrometry proteomics data have been deposited to the ProteomeXchange Consortium via the PRIDE partner repository with the dataset identifier PXD016704 and 10.6019/PXD016704.

For analysis of relative chromatography conditions, the two datasets were reprocessed using the Minora Feature Detector node in Proteome Discoverer 2.4 [25]. One output of the node is the identification of left and right retention times for each chromatographic feature. From these measurements, the average peak widths for the NanoLC experiment was determined to be 48.6 ± 7.2 s. (Supplemental S3) The uHPLC system recorded a peak width of 34.8 ± 0.74 s. (Supplemental S4) The use of the identical dynamic exclusion settings of 30 s appears to have been suboptimal for the NanoLC experiment and corresponds to repeated fragmentation of the same peptides, thereby reducing peptide and total protein identifications. This observation suggests that further optimization of the NanoLC and MS/MS conditions are appropriate due to the wider relative peaks in NanoLC compared to uHPLC using the resources described here. Recent studies that have demonstrated improvements in chromatographic reproducibility when employing between 50 µL/min and 300 µL/min in global proteomic studies, compared to nanoflow chromatography. 

**Figure 1:**
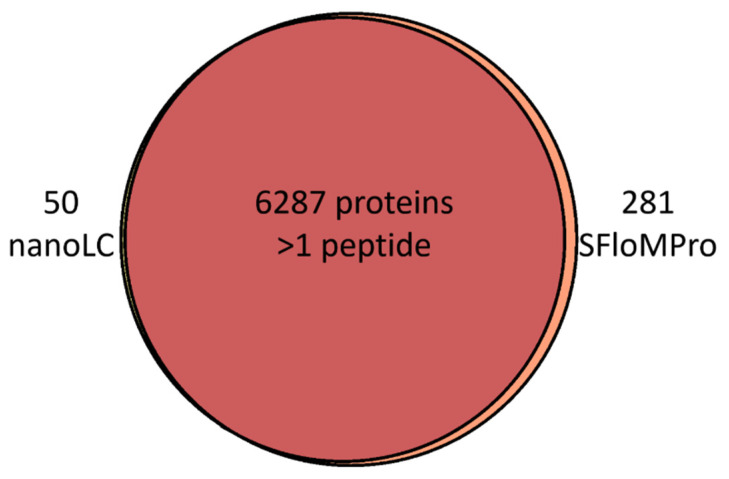
A Venn Diagram showing the overlap in protein identification between experiments for the samples analyzed in this study when filtered to proteins with more than one unique peptide.

**Figure 2:**
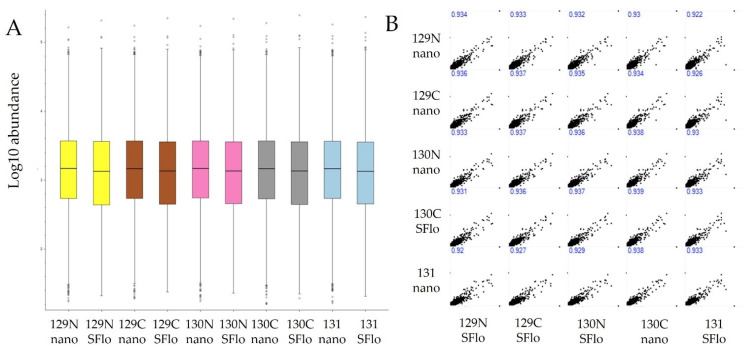
(**A**) Comparative loading plots of the samples from the protein group level. (**B**) Scatter plots demonstrating the comparison of protein abundances for each channel from the two methods.

As shown in Figure 1 the overlap in protein group identifications between the two methods is in high concordance, with proteins greater than 2 peptides per protein shown in the graph. Considering the well-recognized variability in protein identifications by LCMS, these results likely approach the expected theoretical maximum when comparing two separate experiments [26,27]. Furthermore, Figure 2 is a demonstration of the normalized loading plots of each channel. Box plots of the same color represent the total normalized loading of the same channel compared between the NanoLC and SFloMPro experiments and further demonstrates the relative level of concordance between the two sample sets. 

### 3.3. Quantitative Comparisons

**Figure 3:**
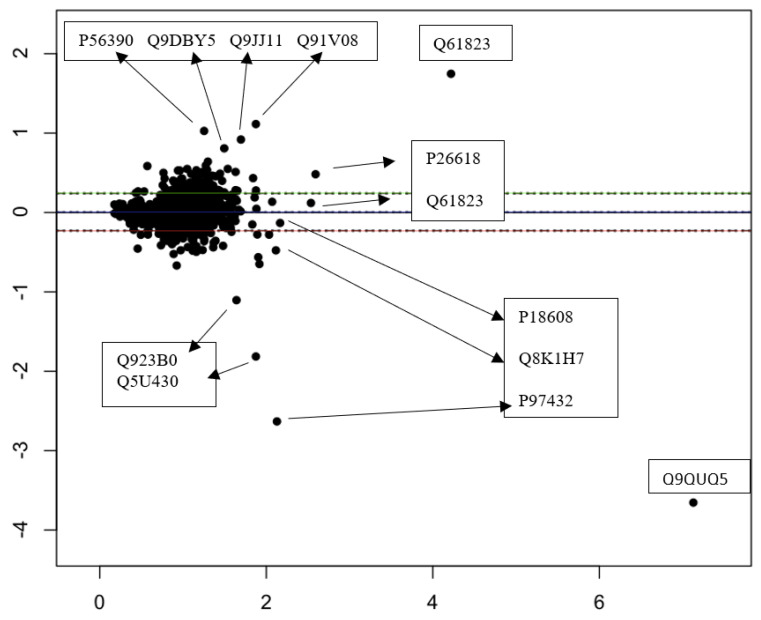
A Bland Altman plot flagging primary outliers for the 128C channel when the two methods are compared by their UniProt SwissProt identifier.

**Figure 4:**
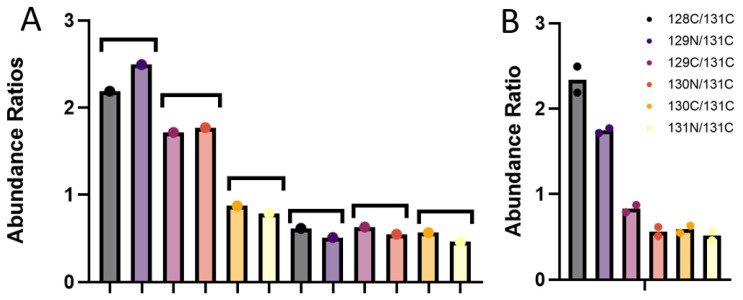
Bar charts demonstrating the quantitative comparison of outlier Q61823. (**A**) Represents the individual ratios from each comparison with the first bar under each bracket representing the ratio from the NanoLC and the second the ratio from the high flow data. (**B**) The data combined demonstrating the relatively small differences between the two analytical methods.

To determine the degree of quantitative overlap between the same samples analyzed by using NanoLC and SFloMPro, correlation analysis at the protein group level was performed on each individual sample between the two experiments. As shown in Figure 2B, we observe a high correlation between protein channels. For a more granular analysis, we constructed Bland Altman plots to flag proteins that were not in quantitative agreement between the two experimental designs. As shown in Figure 3, proteins that were found to be in disagreement quantitatively appeared to be solely related to how quantified peptides were assigned to protein groups. In the example of the most extreme upper outlier Q61823, the difference is flagged by the relative abundance of the 128C channel in the SFloMPro experiment. Due to the high concordance in the quantitative values of the two sets, very small changes in the actual ratios may be flagged as outliers, as in the case between a 2.18 and 2.43 abundance fold change. Figure 4 demonstrates the quantitative values for this protein. 

## 4. Conclusions

In this study, we describe the SFloMPro shotgun proteomics workflow which circumvents technical challenges in the field. By eliminating the NanoLC from the experiment and optimizing analytical flow HPLC, we obtain nearly equivalent data. Furthermore, we find that SFloMPro Proteomics is an economical alternative due increased speed in sample loading and gradient equilibration, when compared to the common commercial NanoLC systems in use in our respective labs. It is worth noting, however, that alternative LC systems can prepare and execute nanoflow LCMS experiments with lower relative overhead time than the systems described here. More sophisticated systems using multiple pumps have historically presented lower relative system overhead as well as the capacity to load multiple analytical columns in parallel [28,29]. One notable advance is the use of inline disposable tips to load chromatography systems with microflow or nanoflow gradients [23,30]. The reduction in the relative overhead of these systems would negate some of the time benefits of SFloMPro, but neither would change the degree of relative complexity in working with nanoflow chromatography, relative to higher flow techniques. Recent work by the Kuster lab has demonstrated the successful application of proteomics using flow rates up to 50 µL/min over thousands of samples with coverage comparable to NanoLC methods [17]. The main differences between this approach and those presented here are in the generation of the MS hardware employed and a relative increase in the complexity of the HPLC plumbing involved. The instruments utilize more modern high field D20 Orbitraps while SFloMPro data demonstrated here uses the older Q Exactive hardware with a D30 Orbitrap which was first released in 2013 [31]. While we fully expect that the application of lower flowrates to improve on the results presented in this study, not all HPLC systems are capable of producing stable microflow gradients. Recent work from Lenčo et al., has painstakingly detailed the optimization of HPLC gradient conditions for label free proteomics. At conditions nearly identical to those of SFloMPro, with the exception of the addition of acetic acid to the running buffers, a fractionated cancer cell line digest obtained 6700 protein identifications when starting with 20 µg of peptide material with marked increases in the chromatographic reproducibility when using analytical flow rates. As in the work described from the Kuster lab, these authors found the highest relative sensitivity when using columns of 1.0 mm in width and flow rates less than 100 µL/min. While we fully expect that the application of lower flowrates to improve on the results presented in this study, not all HPLC systems are capable of producing stable microflow gradients. As such, we believe that the methods presented herein may be more generally applicable to scientists who use standard flow liquid chromatography coupled to high resolution mass spectrometry, particularly when sample concentrations are not strictly limited. 

## Figures and Tables

**Table 1:** An overview of the results of the two experiments described.

Experiment	MS/MS Spectra	PSMs	Peptides	Proteins	Total Run Time (h)
NanoLC	488,715	137,460	65,295	7685	34.50
SFloMPro	359,253	107,552	74,160	8086	25.25

## Data Availability

All data are available via ProteomeXchange [32] with identifier PXD016704.

## Supplementary Materials

The following supporting information can be downloaded at: https://www.mdpi.com/article/10.3390/proteomes10010003/s1. A combined excel sheet containing supplemental information S1–S4 is included with this study.
